# Fructooligosacharides Reduce *Pseudomonas aeruginosa* PAO1 Pathogenicity through Distinct Mechanisms

**DOI:** 10.1371/journal.pone.0085772

**Published:** 2014-01-22

**Authors:** Mercedes Ortega-González, Fermín Sánchez de Medina, Carlos Molina-Santiago, Rocío López-Posadas, Daniel Pacheco, Tino Krell, Olga Martínez-Augustin, Daddaoua Abdelali

**Affiliations:** 1 Department of Biochemistry and Molecular Biology II, Centre of networked Biomedical Research about Hepatic and Digestive Diseases, School of Pharmacy, University of Granada, Granada, Spain; 2 Department of Environmental Protection, Consejo Superior de Investigaciones Científicas, C/Profesor Albareda 1, Granada, Spain; 3 Departments of Pharmacology, Centre of networked Biomedical Research about Hepatic and Digestive Diseases, School of Pharmacy, University of Granada, Granada, Spain; Centre National de la Recherche Scientifique, Aix-Marseille Université, France

## Abstract

*Pseudomonas aeruginosa* is ubiquitously present in the environment and acts as an opportunistic pathogen on humans, animals and plants. We report here the effects of the prebiotic polysaccharide inulin and its hydrolysed form FOS on this bacterium. FOS was found to inhibit bacterial growth of strain PAO1, while inulin did not affect growth rate or yield in a significant manner. Inulin stimulated biofilm formation, whereas a dramatic reduction of the biofilm formation was observed in the presence of FOS. Similar opposing effects were observed for bacterial motility, where FOS inhibited the swarming and twitching behaviour whereas inulin caused its stimulation. In co-cultures with eukaryotic cells (macrophages) FOS and, to a lesser extent, inulin reduced the secretion of the inflammatory cytokines IL-6, IL-10 and TNF-α. Western blot experiments indicated that the effects mediated by FOS in macrophages are associated with a decreased activation of the NF-κB pathway. Since FOS and inulin stimulate pathway activation in the absence of bacteria, the FOS mediated effect is likely to be of indirect nature, such as via a reduction of bacterial virulence. Further, this modulatory effect is observed also with the highly virulent *ptxS* mutated strain. Co-culture experiments of *P. aeruginosa* with IEC18 eukaryotic cells showed that FOS reduces the concentration of the major virulence factor, exotoxin A, suggesting that this is a possible mechanism for the reduction of pathogenicity. The potential of these compounds as components of antibacterial and anti-inflammatory cocktails is discussed.

## Introduction

Strains of *P. aeruginosa* are ubiquitously present in the environment [1], which is due to their capacity to colonize different ecological niches [2], [3] and metabolic versatility [4]. *P. aeruginosa* is an opportunistic pathogen able to infect different animals and plants [5], [6], being a frequent cause of hospital-acquired infections including ventilator associated pneumonia [7] and catheter infections in immuno-compromised patients. *P. aeruginosa* lung infections are the main cause of morbidity and mortality in cystic fibrosis (CF) patients [8]. The bacterium is highly resistant to antibiotic treatment and difficult to eradicate once established in the host [9]. One of the important antibiotic resistance mechanisms is the formation of biofilms [10], hence a great deal of attention has been given to the study of the molecular mechanisms involved in its generation, maturation and dispersal [11], [12]. It has been shown that flagella and type IV pili-mediated motility are required for efficient biofilm formation [13]–[15].

Bacteria use different secretion systems to inject virulence factors into the cytoplasm of eukaryotic cells, leading to bacterial replication within macrophages and, consequently, evasion from the immune system [16]. In Gram-negative bacteria several secretion systems have been characterized, referred to as type I to type VI systems [17], [18]. The type II (T2SS) and type III secretion system (T3SS) secrete the majority of known toxins [19]. They differ in their molecular mechanisms and operate on several substrates. The secretion system type I is an ABC transporter composed of an ABC protein, a membrane fusion protein and an outer membrane protein. This system transports various molecules of diverse nature such as ions, drugs, and proteins [20]. Similarly, type II and V secretion systems generally transport proteins to the surface of the host cell and are involved in the extracellular release of various toxins and hydrolytic enzymes such as exotoxin A, Las A, Las B, protease and elastase [21], [22]. In contrast, the type III secretion system (T3SS) injects proteins, small molecular weight compounds and hydrolytic enzymes into the cytosol of eukaryotic cells [23], which corresponds to a potent virulence mechanism shared by many pathogenic Gram-negative bacteria. This protein injection in turn triggers a cytoskeletal reorganization of the host cell as shown by the inhibition of *P. aeruginosa* internalization upon incubation with cytochalasin D [24], which destroys microfilaments, thereby preventing further uptake of bacteria [16], [23], [25].

A significant number of natural compounds have been found to inhibit bacterial growth, although their mechanism of action remains unclear in most cases [26], [27]. Here we report a study on the activity of the fructo-oligosaccharides (FOS) and inulin on *P. aeruginosa* proliferation. Inulin is a linear polymer formed by 20 to over 60 β-2,1-linked fructose monomers with a terminal glucose residue, whereas FOS are short-chain oligosaccharides with the same structure but a maximal chain length of 2 to 20 monomeric units which are generated by hydrolysis of inulin [28]. Inulin is found in different nutrients such as wheat, onion, garlic and banana [29]. Inulin and FOS are considered prebiotics, based on the observation that they promote the growth of certain beneficial gut bacteria such as bifidobacteria [30], [31], but they have been also found to inhibit the growth of pathogenic bacteria such as *Salmonella typhimurium* [32], *S. enteritidis* [33] *Listeria monocytogenes* or the fungus *Candida albicans* [34]. In addition inulin and FOS have been found to have a beneficial impact on human health, including the stimulation of calcium, iron and zinc absorption [35] and the modulation of local and systemic immune responses [36].

Here we show that the addition of FOS to *P. aeruginosa* PAO1 cultures decreases growth and biofilm formation. This effect appears to be specific for FOS since it was not observed following inulin treatment. In addition, FOS reduces the cytokine response of rat primary monocytes to *P. aeruginosa* infection, an effect considered indirect since this oligosaccharide was found to activate the NF-κB pathway. Attenuated responses are observed also with the virulent *ptxS* mutant strain. Exotoxin A production is lowered by FOS treatment, suggesting that FOS may interfere with exotoxin synthesis and/or secretion. Taken together, our data suggest that FOS may be a useful component of drug cocktails for the treatment of *P. aeruginosa* infections.

## Results

### Differential effect of FOS and inulin on the growth of *P. aeruginosa* PAO1

**Figure 1:**
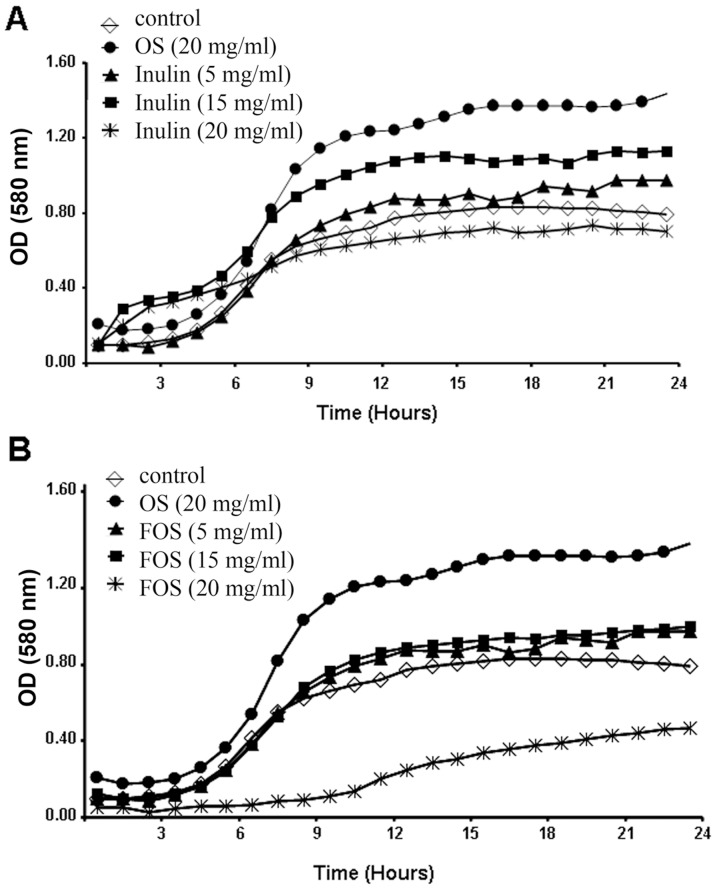
Effect of inulin and FOS on the growth of *P. aeruginosa* PAO1. Growth curves in minimal medium M9 supplemented with 50(A) and FOS (B) are shown. As a control, growth in the minimal medium M9 supplemented with 20 mg/ml of goat milk oligosaccharides (OS) is shown. Growth curves were recorded at 37°C for 24 hours. Representative data from one of three independent experiments with similar results are shown.

As stated in the introduction, a number of natural compounds can either promote or slow down the growth of microorganisms. To assess the effect of inulin and FOS on *P. aeruginosa*, growth curves were recorded in minimal medium M9 supplemented with citrate. Under these conditions only minor changes in the bacterial growth rate and yield were observed in the presence of 5–20 mg/ml inulin. Namely, 5 and 15 mg/ml of inulin resulted in a slight to moderate stimulation of growth, whereas at the concentration of 20 mg/ml a slight reduction was noted (Fig. 1A).

Similarly, in the presence of 5–15 mg/ml of FOS a minor stimulation of bacterial growth was noted (Fig. 1B); however, at the concentration of 20 mg/ml a marked inhibition was observed (Fig. 1B). Since inulin and FOS differ only in the carbohydrate chain length, this parameter appears to be central for antibacterial activity. In comparison, a growth curve was recorded in the presence of 20 mg/ml of control goat milk oligosaccharides (OS), showing promotion of bacterial growth at the same concentration at which FOS inhibits growth. In fact, both FOS and inulin (at 15–20 mg/ml) are able to support bacterial growth when M9 minimal medium is used (data not shown).

### Reduction of biofilm formation in the presence of FOS

**Figure 2:**
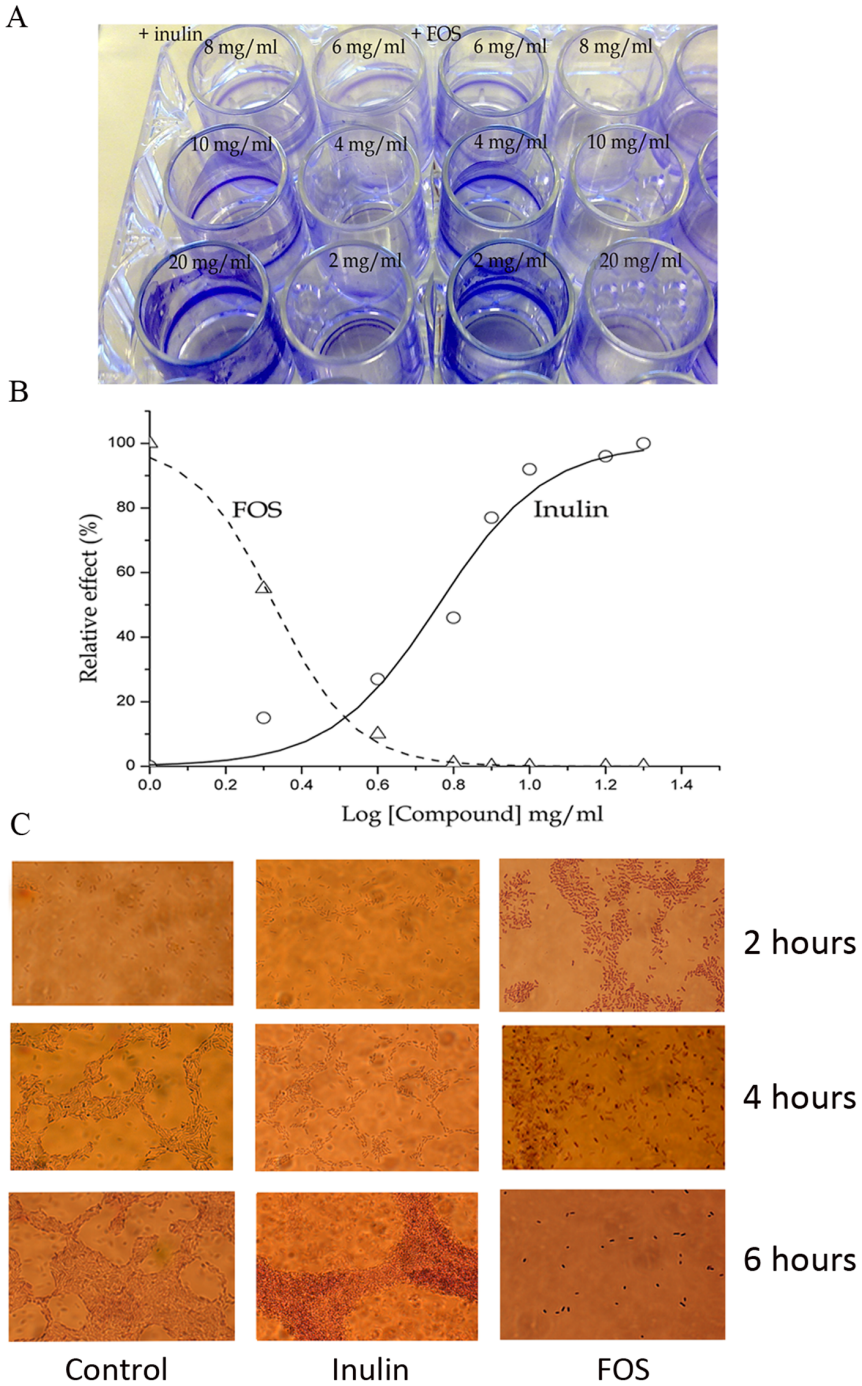
Formation of *P. aeruginosa* biofilm. A) Biofilm formation in the absence and presence of different concentrations of FOS and inulin in 24-well plates. Biofilm formation was monitored in M9 minimal medium supplemented with 0.2% (w/v) glucose and casamino acids and quantified after 6 h. B) The relative amounts of biofilm formation in the experiments shown in Fig. 2A are plotted against the logarithm of inulin/FOS concentration. Data were fitted with the sigmoidal model of the ORIGIN software package to determine EC_50_ values. Data are the average of three independent assays. C) Microscopic inspection of biofilm formation in the absence and in the presence of 20 mg/ml inulin or FOS at 2, 4 and 6 hours.

To assess the influence of FOS and inulin on biofilm formation, *P. aeruginosa* PAO1 was cultured in 24-well plates in the absence or presence of different concentrations of both compounds for 6 hours (Fig. 2A), followed by a quantification of biofilm formation. Fig. 2 B and Fig. S1 show the relative amount of biofilm formation as a function of the inulin/FOS concentration (logarithmic scale). It became apparent that both compounds have opposite effects; whereas inulin stimulated biofilm formation, FOS had a concentration dependent inhibitory effect. Fitting of data (Fig. 2B) resulted in an EC_50_ value of 2.1 mg/ml for FOS (inhibition) and 5.8 mg/ml for inulin (stimulation). Biofilm formation was almost completely inhibited at a FOS concentration of 4–8 mg/ml, which is in sharp contrast with inulin that produced a ∼10-fold enhancement at concentrations up to approximately 10 mg/ml.

Subsequently, biofilms formed in the presence or absence of both compounds (at 20 mg/ml) were observed under the microscope (Fig. 2C). To this end bacteria were grown in M9 minimal medium supplemented with 0.2% (w/v) glucose, 0.4% (wt/v) casamino acids and with inulin or FOS. In control conditions clear biofilm formation was observed after 4 and 6 hours of culture, as expected (Fig. 2C). Similar results were obtained with 20 mg/ml of inulin, but the biofilm was more prominent after 6 h as compared to the control sample. In contrast, while biofilm formation in the presence of FOS was comparable to that in control conditions after 4 hours, it was virtually absent at 6 h (Fig. 2C).

### FOS and inulin have opposite effects on bacterial motility

**Figure 3:**
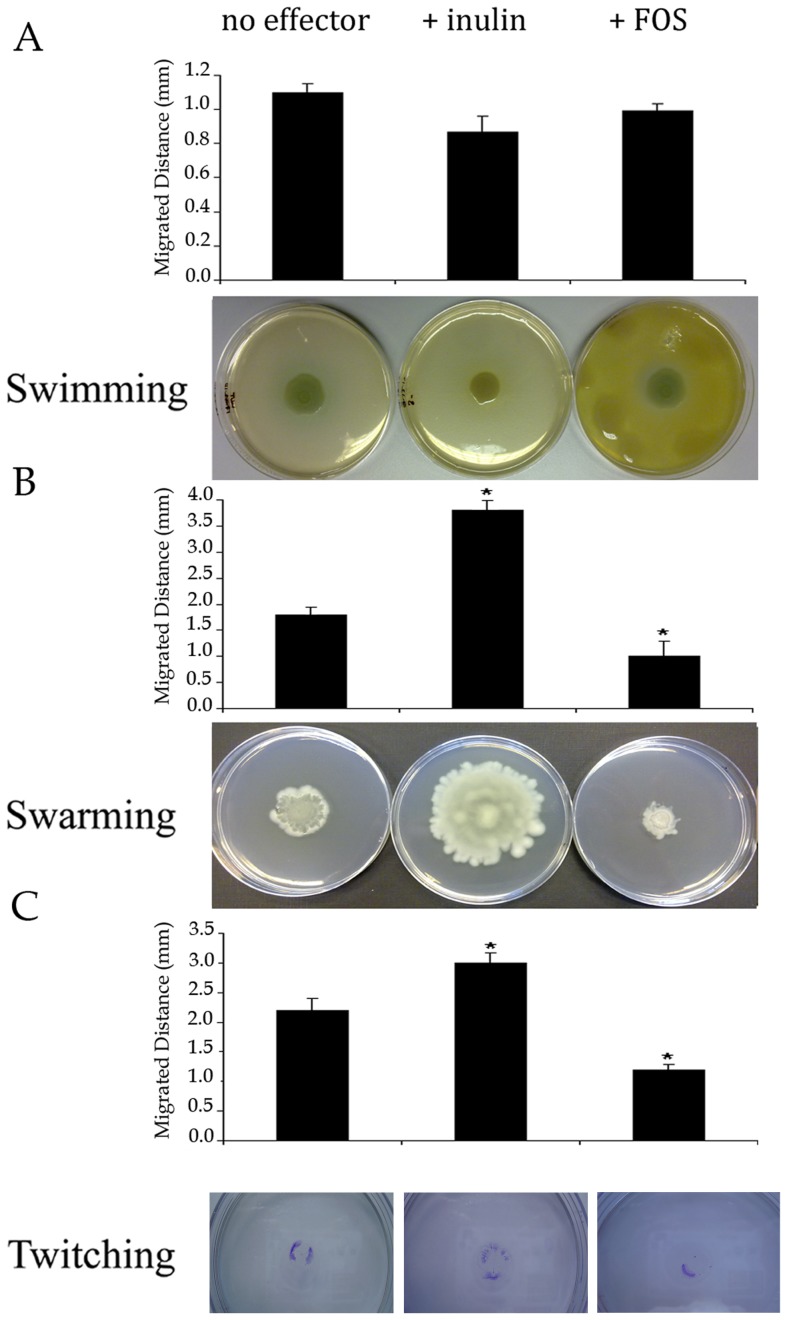
Effects of FOS and inulin on the motility of *P. aeruginosa*. Motility assays were carried out as described in materials and methods. Inulin or FOS at 5 mg/ml was present in the agar plates and in the bacterial suspensions. A) Swimming assays. B) Swarming assays and C) Twitching assays. Average values of the distances of bacterial migration are shown. Data are the average of three independent assays. Values are means ± s.e.m., n = 6; *P<0.05 vs without effectors (ANOVA followed by least significance tests).

*P. aeruginosa* has been shown to exhibit three different types of motility, namely swimming, swarming and twitching [37], [38]. Twitching motility across solid surfaces [39] has been found to be required for biofilm development [13], [40], as well as for a persistent colonization of lungs, and it is associated with virulence in corneal infection models [39], [41]. We have studied the effect of inulin and FOS at 5 mg/ml on *P. aeruginosa* bacterial motility on agar plates and in bacterial suspension (Fig. 3). Neither FOS nor inulin affected swimming behaviour (Fig. 3A). Interestingly, FOS inhibited both swarming and twitching motility, whereas inulin treatment resulted in the opposite effect, i.e. stimulation (Fig. 3B and C).

### Inulin and FOS reduce cytokine secretion in *P. aeruginosa* infected macrophages

Tissue injury or infection results in the recruitment and activation of host immune cells. Macrophages are the first immune cells likely to encounter *P. aeruginosa*. The activation of macrophages is based largely on the recognition of pathogens by molecular pattern receptors, including Toll-like receptors (TLRs), such as TLR4 [16], [42]. Macrophages can internalize and kill bacterial pathogens; however, during *P. aeruginosa* infections their role in pathogen sensing is of primary importance [43]. This in turn causes significant changes in gene expression and the secretion of proinflammatory cytokines IL-6 and TNF-α that recruit inflammatory cells in response to bacterial virulence factors, while IL-10 tends to mitigate this response [44]–[47]. These are among the main signalling mediators released by monocyte/macrophages.

Initial experiments were carried out to establish the experimental conditions for the assessment of the effect of FOS and inulin on cytokine secretion (Fig. S2). Macrophages were incubated with *P. aeruginosa* PAO1 and interleukin 6 secretion was measured at different time intervals (Fig. S2). Maximal secretion was observed after 4 hours of incubation. Therefore, these experimental conditions were used to quantify the effect of FOS/inulin on the secretion of IL-6, IL-10 and TNF-α.

**Figure 4:**
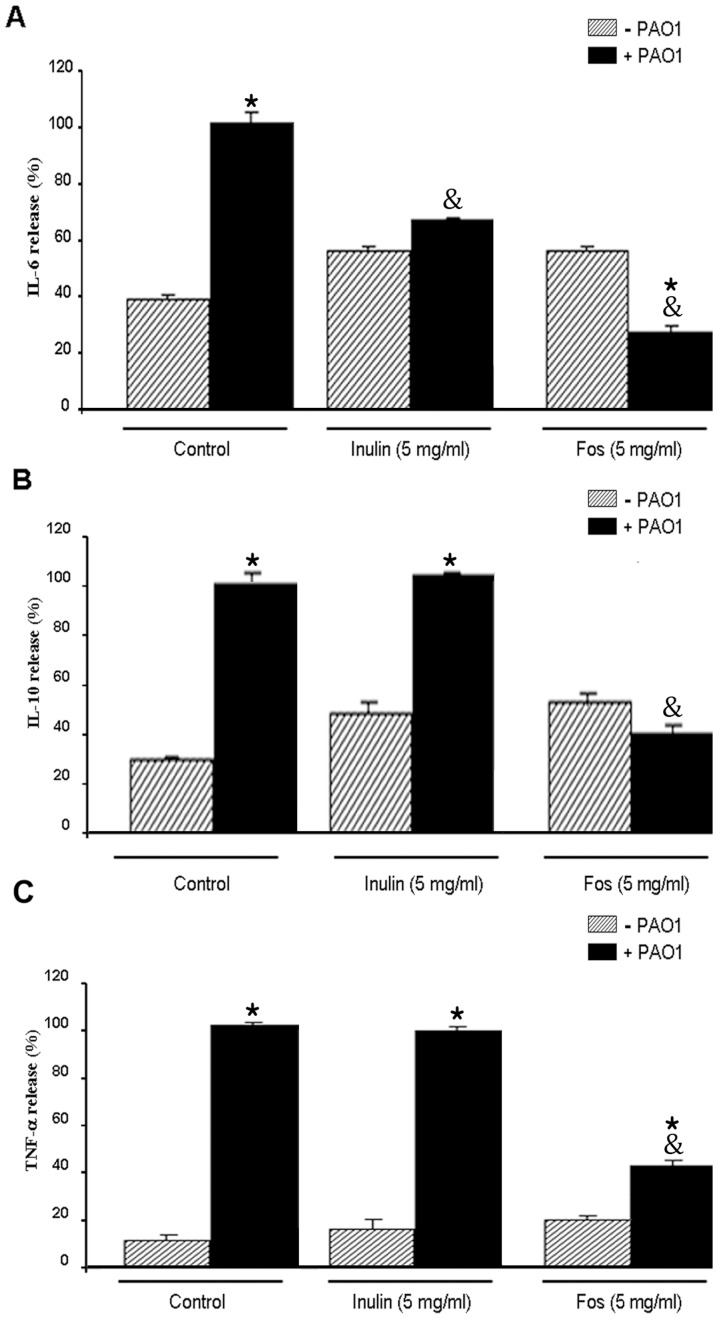
Effect of inulin and FOS on inflammatory response of macrophages against *P. aeruginosa* (WT). Macrophages were incubated with WT *P. aeruginosa* cells (ratio 1/5) for 4 hours in either the absence or the presence of 5 mg/ml FOS and inulin prior to the determination of IL-6 (A), IL-10 (B) and TNF-α secretion (C). Values are means ± s.e.m., n = 6–8; ^*^P<0.05 vs macrophage without bacteria and ^&^P<0.05 vs WT in the absence of inulin or FOS (ANOVA followed by least significance tests).

In the absence of bacteria (Fig. 4, column –PAO1), inulin and FOS had no significant effect on cytokine secretion, although a slight increase was noted. PAO-1 infection caused the expected significant increase in cytokine secretion. This response was markedly attenuated for the three cytokines in the presence of FOS, while inulin caused exclusively a reduction in IL-6 levels (Fig. 4). These data therefore show that the presence of inulin and particularly FOS reduced the inflammatory response of macrophages to bacterial infection. Since FOS/inulin did not reduce the cytokine release in the absence of bacteria, our results suggest that the inhibitory effect of FOS is probably due to a direct interaction with *P. aeruginosa*.

### Activation of the NF-κB signal transduction pathway is downregulated by FOS

**Figure 5:**
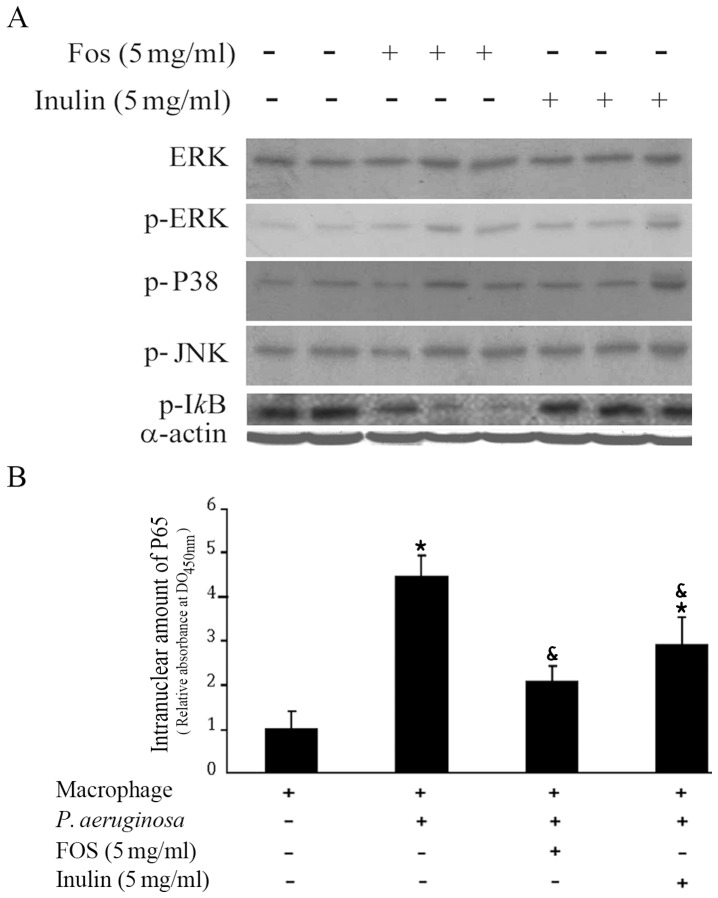
FOS but not inulin reduces the amount of phosphorylated IkB-α. A) Macrophages were infected with *P. aeruginosa* in the absence or presence of either 5 mg/ml FOS or inulin. After 4 h growth Western blots were performed using cell extracts and the corresponding antibodies against ERK (Extracellular Regulated Kinase), p-ERK (the phosphorylated form of Extracellular Regulated Kinase), p-P38 (activated and phosphorylated form of P38 mitogen-activated protein kinases), p-JNK (Jun N-terminal kinases) and after 1 h for p-IκB-α (activated form in the NF-κB canonical pathway). As control, actin was quantified in all samples using an anti-α-actin antibody. Duplicate samples in the absence of added effectors and triplicate samples in the presence of FOS and inulin are shown. B) NF-κB activation was determined by measuring the nuclear translocation of the p65 component, expressed as the OD_450 nm_. ^*^P<0.05 vs macrophage without bacteria and ^&^P<0.05 vs WT in the absence of inulin or FOS (ANOVA followed by least significance tests).

The mitogen-activated protein kinase (MAPK) and the NF-κB signalling pathways are implicated in the production of TNF-α and IL-6 in macrophages [48]. In addition, it has been reported that *P. aeruginosa* infection is associated to stimulation of TLR4 receptors, leading to an activation of the NF-κB pathway [49]. To assess the impact of inulin and FOS on the inflammatory response, the role of the NF-κB and MAPK signalling pathways in the FOS-mediated modulation of macrophages was assessed. One of the ways to activate NF-κB by extracellular stimuli involves the rapid degradation of IκB-α as a consequence of IκB-α phosphorylation at Ser32 by IκB kinase, which corresponds to IKK in the so-called canonical pathway. We studied the effect of inulin and FOS on the activation (phosphorylation) of IκB-α and MAPK, ERK, JNK and p38 by Western blot analysis in macrophages infected with *P. aeruginosa*. As shown in Fig. 5A, neither FOS nor inulin affected the phosphospecific signal of the three MAPK, suggesting that they are not involved in the observed changes. In contrast, FOS but not inulin reduced IκB-α phosphorylation, pointing to a modulation of the NFκ-B canonical pathway.

Moreover, the activation of the NF-κB transcription factor is associated with the nuclear translocation of the p65 component of the complex. To confirm the effect of inulin or FOS on nuclear NF-κB/p65 translocation, p65 was quantified by ELISA in cell nuclei following 1 h activation. In contrast to inulin, FOS (5 mg/ml) effectively reduced NF-κB/p65 translocation (Fig. 5B). Therefore, the effect of FOS is associated with a minor activation of the NF-κB signalling pathway.

### The effect of FOS is observed in *P. aeruginosa* mutant strains with different degrees of virulence

Since the FOS-mediated inhibition of bacterial growth and biofilm formation may reduce virulence, we hypothesized that the response of eukaryotic cells to infection may be modulated by the presence of this oligosaccharide. Subsequent experiments were aimed at assessing the effect of FOS and inulin in two strains of *P. aeruginosa* showing different levels of virulence compared as compared to wild type strain. We used mutants deficient in PtxS and PtxR, two transcriptional regulators that control the expression of the *toxA* gene, encoding the exotoxin A virulence factor [50].

Both, PtxR and PtxS play a role in regulating the activity from the P*_toxA_* promoter [51]. Mutation of *ptxS* increases *toxA* expression by a factor of ∼4, whereas deletion of *ptxR* causes a ∼2-fold reduction [51].

**Figure 6:**
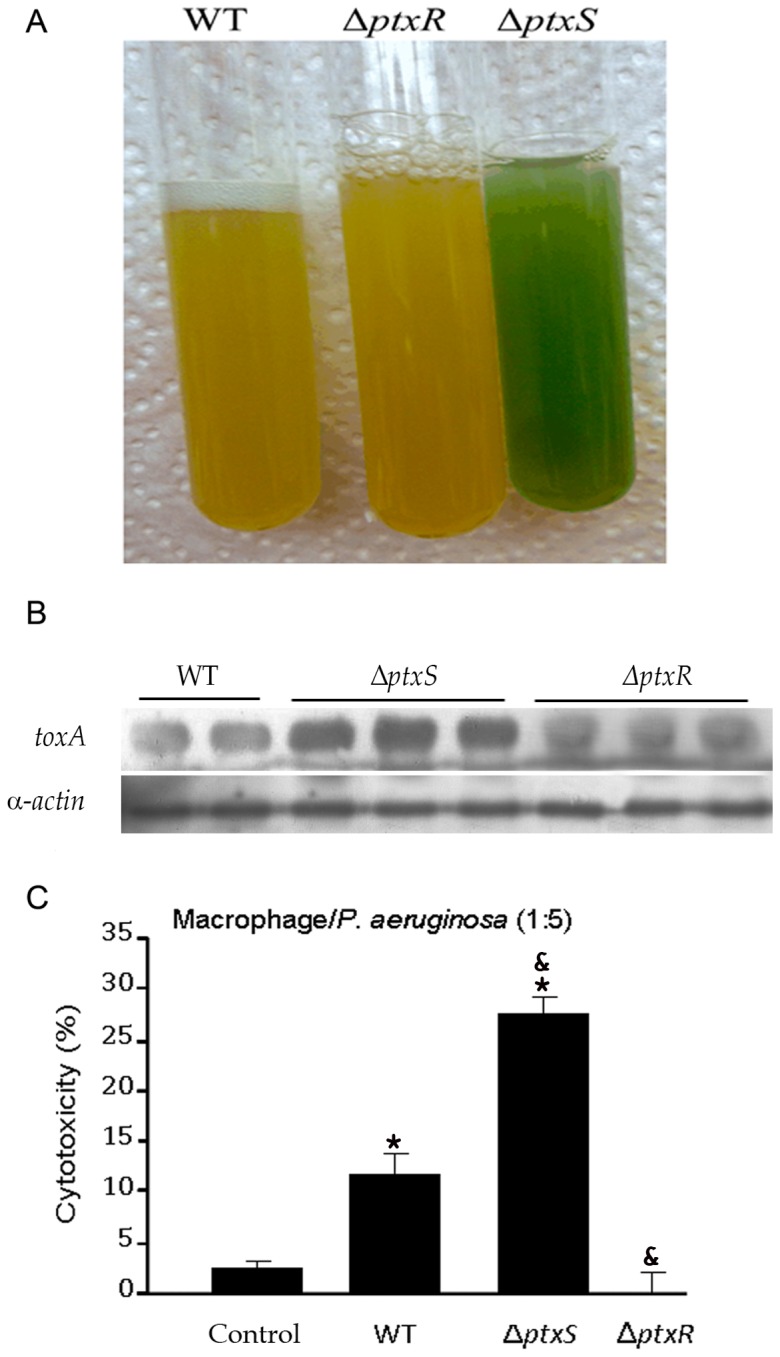
Effect of wild type and mutant *P. aeruginosa* PAO1 on macrophages. A) Cultures of *P. aeruginosa* PAO1 and its mutants deficient in *ptxS* and *ptxR* were grown in M9 Minimum medium supplemented with 50 mM citrate for 24 hours. The blue-green colour is indicative of an increased pyocyanin secretion. B) Western blot determination of exotoxin A concentration secreted by *P. aeruginosa* WT and its isogenic mutants *ptxS* and *ptxR*. C) The macrophage mortality induced by WT *P. aeruginosa* and its *ptxS* and *ptxR* mutants was measured by the total release of cytoplasmic lactate dehydrogenase (LDH). ^*^P<0.05 vs macrophage without bacteria and ^&^P<0.05 vs macrophage with WT (ANOVA followed by least significance tests).

The increase in toxicity of the *ptxS* mutant (Fig. 6A) is reflected in the colour of bacterial cultures due to the increased production of the bright blue-green siderophore pyocyanin, an important virulence factor of fluorescent Pseudomonads [52]–[54]. In contrast the colour of the *ptxR* mutant is similar to that of the wild type strain. Further, anti-exotoxin A western blot showed that *ptxS* mutant produces significantly more exotoxin A than the *ptxR* mutant (Fig. 6B). Subsequently, the cytotoxic activity of *P. aeruginosa* on macrophages was analysed 4 hours after infection, using the Cytotox 96® non-radioactive cytotoxicity assay kit. As expected, cytotoxicity was highest for the *ptxS* mutant (Fig. 6C) where approximately 25% of cells died, followed by the wild type strain (10% of cell death) and the *ptxR* mutant for which no toxicity was detected (Figure 6C). The lactase dehydrogenase (LDH) is a marker for cytotoxicity. We have determined the LDH levels of 0.1–0.2 mU/µl in the absence of bacteria but in the presence of FOS or inulin (data not shown). Both compounds did not induce any significant changes in the LDH levels, indicating that they are not cytotoxic to macrophages under the conditions used (data not shown).

**Figure 7:**
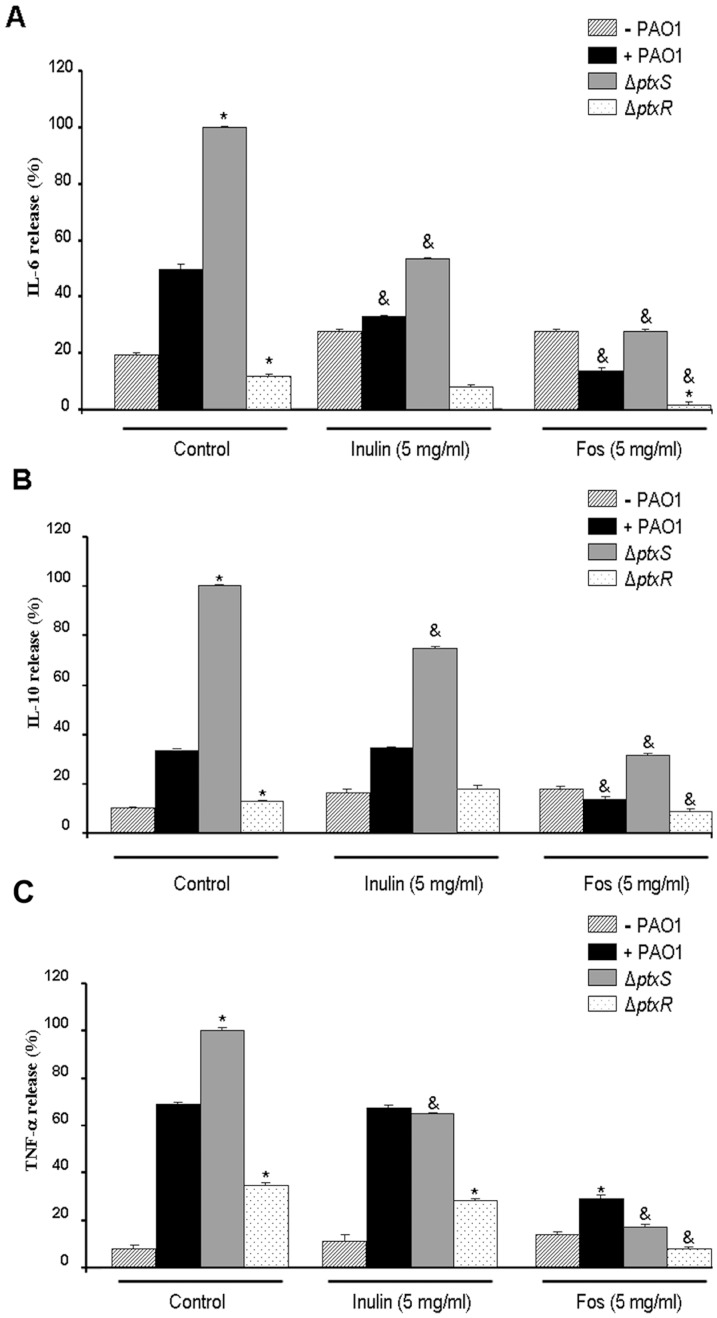
Effect of inulin and FOS on inflammatory response of macrophages against WT *P. aeruginosa* and its isogenic mutants *ptxS* and *ptxR*. Macrophages were incubated with WT and mutant *P. aeruginosa* cells (ratio 1/5) for 4 hours in either the absence or the presence of 5 mg/ml FOS and inulin prior to the determination of IL-6 (A), IL-10 (B) and TNF-α secretion (C). Values are means ± s.e.m., n = 6–8; *P<0.05 vs WT in the absence of inulin or FOS; ^&^P<0.05 vs *ptxS* mutant without effectors (ANOVA followed by least significance tests).

As expected, the cytokine secretory response followed the same pattern, being higher with the *ptxS* mutant, followed by the wild type and lower for the *ptxR* mutant (Fig. 7) (Data from Fig. 4 are included for comparison) [51]. For all three bacterial strains analysed, FOS caused a very pronounced reduction (Fig. 7), while inulin caused a more moderate reduction. Interestingly, the FOS/inulin mediated reduction was more pronounced in the *ptxS* mutant than in WT strain, suggesting that virulent strains may be more sensitive to the effect of fructose oligosaccharides.

**Figure 8:**
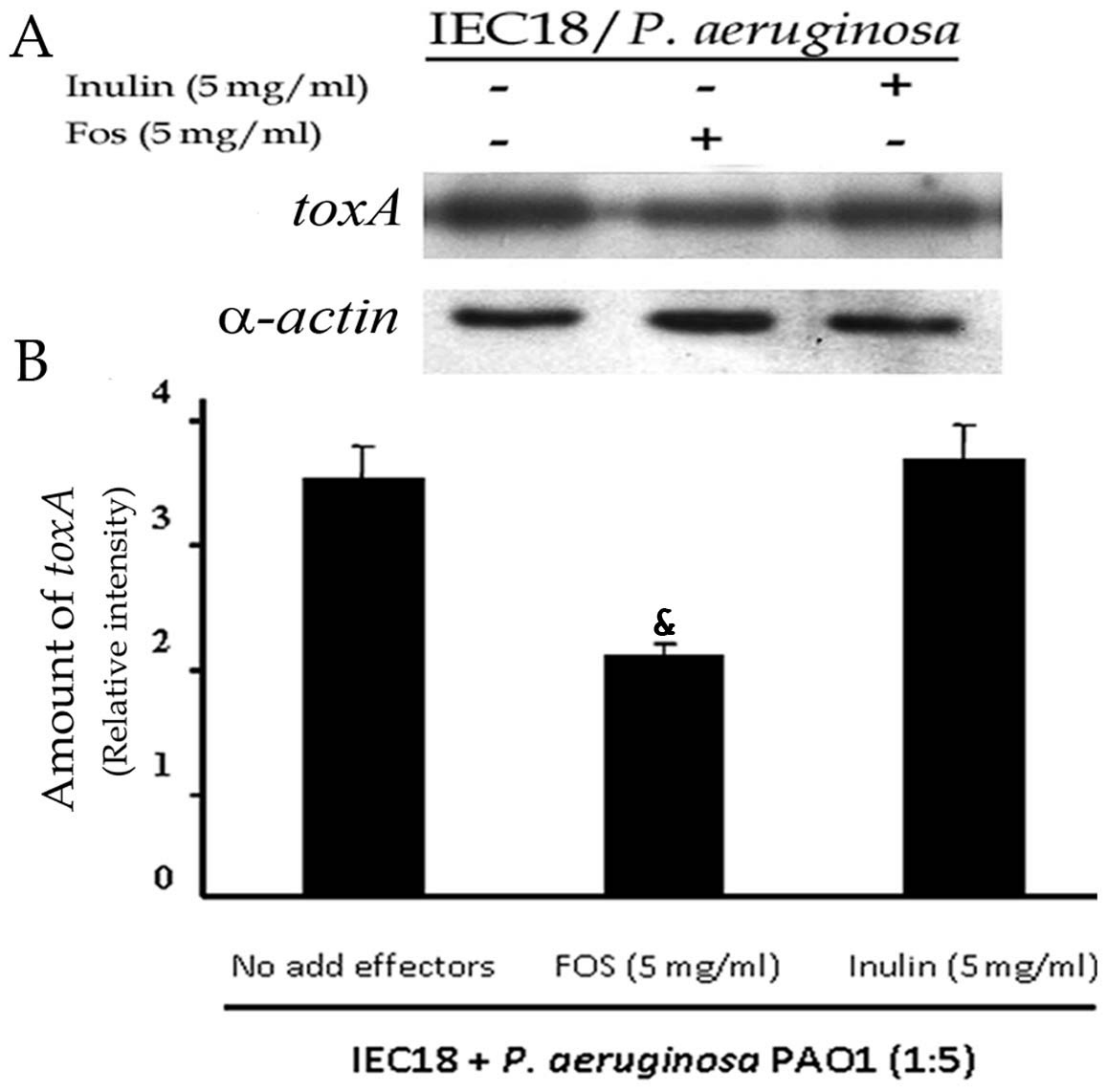
FOS reduces exotoxin A expression. A) Western blot determination of the cellular concentration of exotoxin A in IEC18 cells following co-culture with *P. aeruginosa* in the presence and absence of FOS and inulin. B) Densitometric analysis of above data. Exotoxin A densities were corrected with those obtained for α-actin. Values are means ± s.e.m., n = 3; ^&^P<0.05 vs macrophage/WT without inulin or FOS (ANOVA followed by least significance tests).

*P. aeruginosa* employs a number of systems to secrete proteins which play different roles during infection. To analyse the role of FOS or inulin on *P. aeruginosa* virulence, exotoxin A was quantified in supernatants of bacterial co-cultures with eukaryotic cells as well as within eukaryotic cells. To achieve a good separation of eukaryotic cells from bacteria, we used the rat small intestinal cell line IEC18 that grows on surfaces and exhibits inflammatory responses [55], [56]. Bacteria are removed by a washing step with fresh PBS solution, leaving an intact IEC18 cell monolayer containing infecting *Pseudomonas*. Anti-exotoxin A western blot analysis showed that the addition of FOS and inulin to eukaryotic cells did not alter exotoxin A levels present in the culture medium (data not shown). In contrast, FOS was found to reduce intracellular exotoxin A levels in IEC18 cells co-cultured with *P. aeruginosa*, whereas no significant change was observed in the presence of inulin (Fig. 8A and B). These data suggest that the type II-dependent exotoxin A secretion from *P. aeruginosa* to the cell cytosol is inhibited by FOS, presumably limiting its virulence. Because we cannot rule out the presence of extracellular, cell adherent bacteria in the sample, it may be possible that FOS also downregulates exotoxin A in extracellular *Pseudomonas*.

## Discussion

Prebiotics are defined as compounds that have beneficial effects on humans by altering the intestinal microbiota in a manner that is beneficial to health. The mechanism of their action is not clear, but it is thought to involve preferential utilization of oligosaccharides by host-friendly bacterial species such as bifidobacteria or lactobacilli, indicating that prebiotic substances might have the capacity to protect against infections and reduce the presence of clinically relevant pathogens in the faecal flora [57], [58]. Another proposed mechanism involves enhanced bacteriocin secretion by lactobacilli [59], which in turn facilitates the incorporation of bacteria into a niche and inhibits the invasion of competing strains or pathogens, leading ultimately to a modulation of the microbiota and of the host immune system.

Therefore, the inhibition of pathogens by prebiotics is thought to be largely due to indirect effects [60]. However, antimicrobial properties have been described for a number of oligosaccharides [61]. To our knowledge this is the first report showing that FOS, one of the most studied and used prebiotics, has specific effects on *P. aeruginosa* PAO1. We were able to show that FOS (1) inhibits *P. aeruginosa* growth, biofilm formation and motility; (2) limits the *P. aeruginosa* evoked NF-κB dependent cytokine secretion in macrophages; and (3) decreases exotoxin A levels in *P. aeruginosa* infected IEC18 cells.

These effects have also been observed for inulin, but in general the magnitude of the changes induced by FOS was superior to that of inulin. This indicates that the length of the oligosaccharide chains is an essential determinant for the magnitude of the biological activities observed. This is exemplified by growth inhibition: both FOS and inulin can be used as carbon and energy source by *P. aeruginosa* and it is therefore not surprising to see a growth stimulation in the presence of up to 10 mg/ml of both compounds (Fig. 1 and data not shown). However, at a concentration of 20 mg/ml inulin had a very modest effect on growth which contrasts with FOS that caused a very significant growth inhibition. These results are consistent with a biphasic response of *P. aeruginosa* to FOS/inulin: growth is stimulated at low concentrations of inulin and then inhibited to a certain extent; the same response is observed with FOS, but growth inhibition is clearly more pronounced. The control goat milk oligosaccharides did not produce any inhibition at similar concentrations, underlining the specificity of action of FOS.

The variety of cellular processes modulated by FOS was a surprising finding and it is tempting to speculate that glycoside receptors may be involved in the recognition of FOS and that these receptors signal via different cascades modulating different cellular processes. Such a mechanism would be comparable to that for the sensing of other antimicrobial glycosides [62]. Otherwise, biofilm formation is a major mechanism that confers bacterial resistance and biofilm induced antibiotics tolerance is of major clinical importance [63]. Currently significant research efforts are being made to identify compounds that interfere with biofilm formation, maturation and dispersion and to explore their effect in infection models [64]. Here we show that FOS and inulin have opposite effects on biofilm formation. While inulin caused a stimulation, a dramatic reduction was observed in the presence of FOS (Fig. 2 and Fig. S1). Interestingly, FOS did not appear to affect the early stages of biofilm formation since no significant changes were observed after 4 hours growth (Fig. 2C). In turn, a dramatic reduction was seen after 6 hours (Fig. 2C), suggesting that FOS interferes with later stages of biofilm formation or triggers its dispersal. Further experiments to elucidate this point are underway.

We hypothesized that these effects may alter *P. aeruginosa* virulence. In order to test this hypothesis, we infected rat primary monocyte cultures with PAO1 and measured cytokine release in the presence and absence of oligosaccharides. Monocytes display an enhanced release of cytokines in response to infection, which was shown to be chiefly dependent on NF-κB activation and p65 nuclear translocation. These are early changes associated with modest cytotoxicity due to the short incubation time. Remarkably, treatment with FOS attenuated significantly all the steps of this response, i.e. secretion of IL-6, IL-10 and TNF-α, IκB-α phosphorylation and p65 nuclear translocation (Figs. 4 and 5). Inulin in contrast had a significantly more moderate effect. In the absence of bacteria neither FOS nor inulin caused an effect on cytokine secretion and their long-term effect on macrophages (and intestinal epithelial cells) is an activation of the NF-κB pathway [65] and thus contrary to the oligosaccharide reduction of pathway activity. Thus it follows that the inhibited NF-κB response in this case is likely to be of indirect nature, i.e. borne out of a reduced stimulation by *P. aeruginosa* infection, confirming our hypothesis. This may also explain why inulin failed to inhibit IκB-α phosphorylation, since it is also capable of activating this signalling pathway; however, it is unclear why inulin decreases IL-6 and IL-10 secretion. Our data suggest that MAPK is not involved in this process.

We then investigated potential links between FOS and other determinants of *P. aeruginosa* virulence, such as the PtxS and PtxR regulators that modulate exotoxin A expression (Fig. 6B). Mutation of the corresponding genes showed the expected alterations in cytokine release and cytotoxicity in infected monocytes, respectively, confirming previous observations [51]. These changes correlated with *toxA* expression, since the highest cytokine secretion/*toxA* transcription was observed in the *ptxS* mutant, followed by the WT strain and the *ptxR* mutant [51]. It is therefore likely that exotoxin A plays a role in stimulating cytokine secretion in the host cell. The main finding of this series of experiments is that FOS exerts its modulatory effects also on the more virulent *ptxS* mutant. Of note, the effect of inulin was somewhat increased compared to that on the wild type, although always lower than that of FOS. Our results therefore suggest that FOS effectively reduces the virulence of 3 different strains of *P. aeruginosa*. Modulation of exotoxin A expression or transport can probably be associated with this effect.

*P. aeruginosa* is an opportunistic pathogen and can infect immunocompromised individuals at different sites such as the respiratory tract, intestine, skin, urinary tract, and wounds. The severity of the opportunistic infection depends to a great extent on the virulence factors expressed by the bacterium, which in turn influence cytotoxicity and antibiotic resistance. As a result *P. aeruginosa* infections are notoriously difficult to treat. Several classes of antibiotics including cephalosporins, penicillins, carbapenems, quinolones and aminoglycosides are currently been used to combat *P. aeruginosa* clinically, but specific sensitivity testing is mandatory [66]. Our data suggest that FOS may be a useful component of a drug cocktail to combat *P. aeruginosa* infection. Alternatively, it appears also plausible to use FOS in a prophylactic context to prevent gastrointestinal infections. Oral FOS supplements are currently been used to prevent gastrointestinal infections [67], which implies that the oral route may be also valid to administer FOS to fight *Pseudomonas* infection. However, any clinical application will require an extensive study of the potential effects of FOS on the human body which are issues that are to be addressed in the future.

## Materials and Methods

### Animals

Wistar rats (200–250 g) obtained from the Animal Service Laboratory of the University of Granada (Spain) were used, housed in macrolon cages, and maintained in air-conditioned animal quarters with a 12-h light-dark cycle. Rats were given free access to tap water and food. This study was carried out in accordance with the Directive for the Protection of Vertebrate Animals used for Experimental and other Scientific Purposes of the European Union (86/609/EEC) and the animal protocol used has been approved by the ethics committee of the Granada University.

### Bacterial strains used in this study

*P. aeruginosa* PAO1 and its mutants deficient in *ptxS* and *ptxR* obtained through insertion of streptomycin and tetracycline cassettes, respectively [51], were grown in LB medium or M9 minimal medium (Na_2_HPO_4_: 6 g/l; KH_2_PO_4_: 3 g/l; NaCl: 0.5 g/l; NH_4_Cl: 1 g/l, 1 mM MgSO_4_, 0.3 mM CaCl_2_ and 0.2 ml/l of 1% ferric ammonium citrate) [68]. When required, antibiotics were added to the culture medium to reach a final concentration of 50 µg/ml ampicillin, 50 µg/ml streptomycin and 30 µg/ml tetracycline.

### Chemicals

Inulin and FOS were provided by BENEO-Orafti (Tienen, Belgium). FOS and inulin were kindly provided by BENEO Orafti® (Tienen, Belgium). Orafti® GR (inulin) is a food ingredient consisting mainly of chicory root inulin, a mixture of oligo- and polysaccharides which are composed of fructose units linked together by β(2-1) linkages. Almost every molecule is terminated by a glucose unit. The total number of fructose or glucose units ( = Degree of Polymerization or DP) of chicory inulin ranges mainly between 2 and 60. Orafti® P95 oligofructose (FOS) is produced by the partial enzymatic hydrolysis of chicory-derived inulin, consisting mainly of molecules with DP between 2 and 8. Solutions were made at 200 g/l in M9 minimal medium and, in the case of eukaryotic cell cultures, in Dulbecco's Modified Eagle Medium (DMEM) containing fetal bovine serum (10%), 2 mM L-glutamine, and 2.5 mg/mL amphotericin B, all provided by Sigma. Solutions were filtered using 0.22 µm cut-off filters and aliquots were stored at −20°C. The goat milk oligosaccharides (OS) were obtained according to the method described in [69]. A product containing >80% of the original oligosaccharide content, only 5% (w/w) of lactose and virtually salt free, was obtained and used to carry out all the experiments.

### Effects of inulin and FOS on *P. aeruginosa* growth

Individual colonies of *P. aeruginosa* PAO1 were picked from the surface of freshly grown LB plates and grown overnight in M9 minimum medium (supplemented with 5 mM of citrate) at 37°C. The overnight culture was diluted with fresh M9 minimum medium to an OD_660 nm_ of 0.05 and 96 well flat-bottomed polystyrene microtiter plates were filled with 180 µl of this cellular suspension. Then, 20 µl of either inulin or FOS were added to reach final concentrations of 5, 15 and 20 mg/ml. Plates were incubated at 37°C under continuous agitation in a Bioscreen C MBR analyser FP-1100-C (OY Growth Curves Ab Ltd., Raisio, Finland). The turbidity was measured using a wideband filter at 420–660 nm every 60 minutes over a 24 h period. The measurements at 580 nm were used to generate growth curves. Some cultures were carried out with the same medium without citrate.

### Semiquantitative determination of biofilm formation

Semiquantitative determination of biofilm formation was performed as described [70]. Experiments were conducted in 24 well flat-bottomed polystyrene microtiter plates in M9 minimal medium supplemented with 0.2% (w/v) of glucose and 0.4% (w/v) casamino acids in the absence and in the presence of different concentrations (up to 20 mg/ml) of inulin or FOS. Biofilm formation was quantified after 6 h by staining with crystal violet following the method described in [71]. The structure of biofilms was observed under contrast-phase microscopy using a Zeiss Axioscope fluorescence microscope coupled to a Nikon DSS-Mc CCD camera and a 100-fold magnifier. Data reported are means from two independent experiments each conducted in quadruplet repeats.

### Motility assays

Assays were carried out to determine the effect of inulin and FOS (each at a concentration of 5 mg/ml) on swimming, twitching and swarming. In all assays these compounds were added at identical concentrations to the bacterial suspension in the plates. For swimming assays bacteria were placed with the help of a sterile tooth-pick at the centre of plates containing a 5 mm layer of LB medium with 0.3% (w/v) Bacto agar, 0.2% casamino acids (w/v) and 30 mM glucose. Plates were incubated at 37°C for 24 h and the radial diffusion of bacteria, due to swimming, was measured. To monitor twitching motility bacteria were placed with a toothpick into a 2 mm thick layer containing 1.5% (w/v) Bacto agar, 0.2% (w/v) casamino acids and 30 mM glucose. After incubation for 24 h at 37°C, the expansion of bacteria on the plate was observed. For swarming assays 5 µl of an overnight culture of bacteria were placed into the centre of swarm plates, which are made of 0.5% (w/v) Bacto agar supplement with 0.2% (w/v) casamino acids and 30 mM glucose. Plates were incubated at 37°C for 24 h, followed by an inspection of the bacterial surface movement [72]. All the motility assays were performed in triplicates.

### Macrophage cell isolation by magnetic activated cell sorting

Female Wistar rats were sacrificed by cervical dislocation and the spleen was extracted aseptically. Cell suspensions were obtained by disrupting the tissues between dissecting forceps in medium. After centrifugation (1500×rpm/5 min), cells were cleared of erythrocytes by resuspension in hypotonic lysis buffer (15 mM NH_4_Cl, 10 mM KHCO_3_, 0.1 mM Na_2_EDTA, pH 7.3) for 30 min on ice. Mononuclear cells were washed and resuspended in MACs buffer (PBS containing 0.5% (w/v) BSA, 2 mM EDTA, pH 7.2). To obtain a monocellular suspension, cells were passed through 70 µm nylon mesh prior to magnetic labelling and subsequently isolated by negative selection. To remove lymphocytes, CD161.1-biotin (1∶200), CD45RA-PE (1∶200) and CD3 (1∶150) (Biosciences), were added and incubated at 4°C for 30 min. Cells were washed and sedimented by centrifugation at 1500×rpm for 5 min. After resuspension in MACs buffer, 25 µl of each antibiotin Microbeads and anti-PE-microbeads (Miltenyi Biotec), were added and the resulting suspension incubated at 4°C during 30 min. Cells were washed, centrifuged and dissolved in DMEM medium (Dulbecco's Modified Eagle Medium). CD161.1+, CD45RA+ and CD3+ cells were discarded using an LD column (Miltenyi Biotec). Macrophages in the flow-through were centrifuged at 1500×rpm for 5 min and resuspended in Dulbecco's Modified Eagle Medium (DMEM Sigma ®) supplemented with 10% FBS (sigma), 2.5 mg/L amphotericin B and 2 mM L-glutamine.

### Measurement of inulin- and FOS-induced changes in cytokine secretion from macrophages following infection by *P. aeruginosa* PAO1

For the determination of cytokine levels the macrophage suspensions (10^6^ cells/ml DMEM medium) were co-cultured with *P. aeruginosa* and incubated with 5 mg/ml FOS or inulin for 4 hours. Following centrifugation at 4°C and 10.000×rpm for 5 min, the resulting supernatants were frozen at −80°C. Aliquots were thawed and cytokine levels determined using ELISA-based kits (BD Biosciences, Erembodegem, Belgium) following the protocol provided by the manufacturer. In addition, macrophage cells were used for the quantification of phosphorylated IκB-α and MAP kinases by Western blot determination as described below.

### Western blot

For the detection of ERK, p-ERK, p-P38, p-JNK and phosphorylated IκB-P, cells were homogenized in lysis buffer (PBS containing 0.1% (w/v) SDS, 0.1% (w/v) sodium deoxycholate, 1% (v/v) Triton X-100) with protease inhibitor cocktail (Sigma) 1∶100 (v/v). Subsequently, homogenates were sonicated and centrifuged 7000× g for 5 min at 4°C. Protein concentrations were determined using the Bicinchoninic acid assay [73]. Samples were boiled in 5× Laemmli buffer (220 mM Tris, 312 µM SDS, 50% (v/v) glycerol, 1% (v/v) 2-mercaptoethanol, 22.5 mM EDTA, pH 6.8, containing traces of bromphenol blue) for 5 min, separated by SDS-PAGE, electroblotted to PVDF membranes (Millipore, Madrid, Spain), and exposed to the primary antibodies against ERK, p-ERK (both from Sigma), p-P38, p-JNK and phosphorylated IkB-P, respectively (all three from Cell signalling, Danvers, MA). Prior to exposure to the secondary IgG Peroxidase antibody (anti-mouse IgG for ERK and p-ERK, anti-rabit IgG for p-P38, p-JNK and p-IkB, (Sigma) the bands were visualized by enhanced chemiluminescence (PerkinElmer, Waltham, MA) and quantified with the NIH software (Scion Image).

### Determination of the NF-κB p65 subunit in macrophages nuclear extracts

Macrophages were co-cultured with *P. aeruginosa* with a 1∶5 ratio and incubated with FOS (5 mg/ml) or inulin (5 mg/ml). After 1 h, nuclear extracts were obtained using a nuclear extract kit (Active Motif, Belgium) and NF-κB activation was determined by quantifying the p65 component using a TransAM kit following the protocols recommended by the manufacturers (Active Motif, Belgium).

### Cytotoxicity Assays

*Measurement of P. aeruginosa induced cytotoxicity in macrophages:* Macrophages were incubated with *P. aeruginosa* with a ratio of 1∶5 for 4 hours and the percentage of cytotoxicity was determinate using cytotox 96 non-radioactive cytotoxicity assay kit following the protocols recommended by the manufacturers (Promega) which evaluates cytotoxicity by assessing the total release of cytoplasmic lactate dehydrogenase (LDH), by the calorimetric detection, into culture medium as a consequence of damaged cell membranes [74].

### Determination of exotoxin A concentration in rat IEC18 cells infected by *P. aeruginosa* PAO1

IEC18 cells were cultured in 6 well plates. At confluence IEC18 cells were infected with *P. aeruginosa* at a ratio of 5 bacterial cells per eukaryotic cell in the absence and in the presence of 5 mg/ml FOS or inulin. Plates containing cells were washed 3 times with PBS and incubated with gentamicin at 100 µg/ml for 1 h to eliminate bacteria. Subsequently, plates were washed 3 times with PBS prior to cell collection using RIPA buffer [25 mM Tris–HCl, pH 7.2, 150 mM NaCl, 0.1% sodium deoxycholate and 0.1% sodium dodecyl sulfate (SDS)] containing a protease inhibitor cocktail (Sigma). Proteins were extracted for Western blot analysis, as described above. Western blot were carried out as outlined above using the Exotoxin A antibody (Sigma) at a 1∶2.000 dilution. Following overnight incubation with the primary antibody the membrane was incubated with the secondary IgG Peroxidase anti-rabbit antibody (Sigma) at a 1∶3.000 dilution for two hours. The bands were detected by enhanced chemiluminescence (PerkinElmer, Waltham, MA) and quantified with NIH software (Scion Image).

### Statistical analysis

All results are expressed as means with the corresponding standard deviations. Differences among means were analysed for statistical significance by a one-way ANOVA analysis and a posteriori least significance test. All analyses were carried out using the SigmaStat 2.03 program (Jandel Corporation, San Rafael, CA). Concentration-response curves were fitted to a logarithmic curve when possible with Origin 7.0 (OriginLab Corporation, Northampton, MA). Differences were considered significant at P<0.05.

## Supporting Information

Figure S1 **The OD at 560 nm of crystal violet (CV) stained and resuspended bacteria from biofilm are given.** Shown are means and standard deviations with n = 3–6; *P<0.05 vs WT in the absence of inulin or FOS (ANOVA followed by least significance tests). The densitometric analysis of experiments is shown in Fig. 2A.
(TIF)

Figure S2 **Measurement of interleukin-6 secretion of macrophages in the presence of WT** ***P. aeruginosa*** **PAO1 and its** ***ptxS*** **and** ***ptxR*** **mutants.** The macrophage/bacteria ratio was of 1∶5. Experimental conditions involving incubation for 4 hours were subsequently used to assess the effect of FOS and inulin on interleukin secretion as reported in Fig. 4 and 7. Values are means ± s.e.m., n = 3; *P<0.05 vs macrophage without bacteria and ^&^P<0.05 vs macrophage with WT (ANOVA followed by least significance tests).
(TIF)
