# Supplementary figures and images for: Fructooligosacharides Reduce Pseudomonas aeruginosa PAO1 Pathogenicity through Distinct Mechanisms
Source: PLoS One. 2014 Jan 22;9(1):e85772. doi: 10.1371/journal.pone.0085772 (PMC3899050; doi:10.1371/journal.pone.0085772)

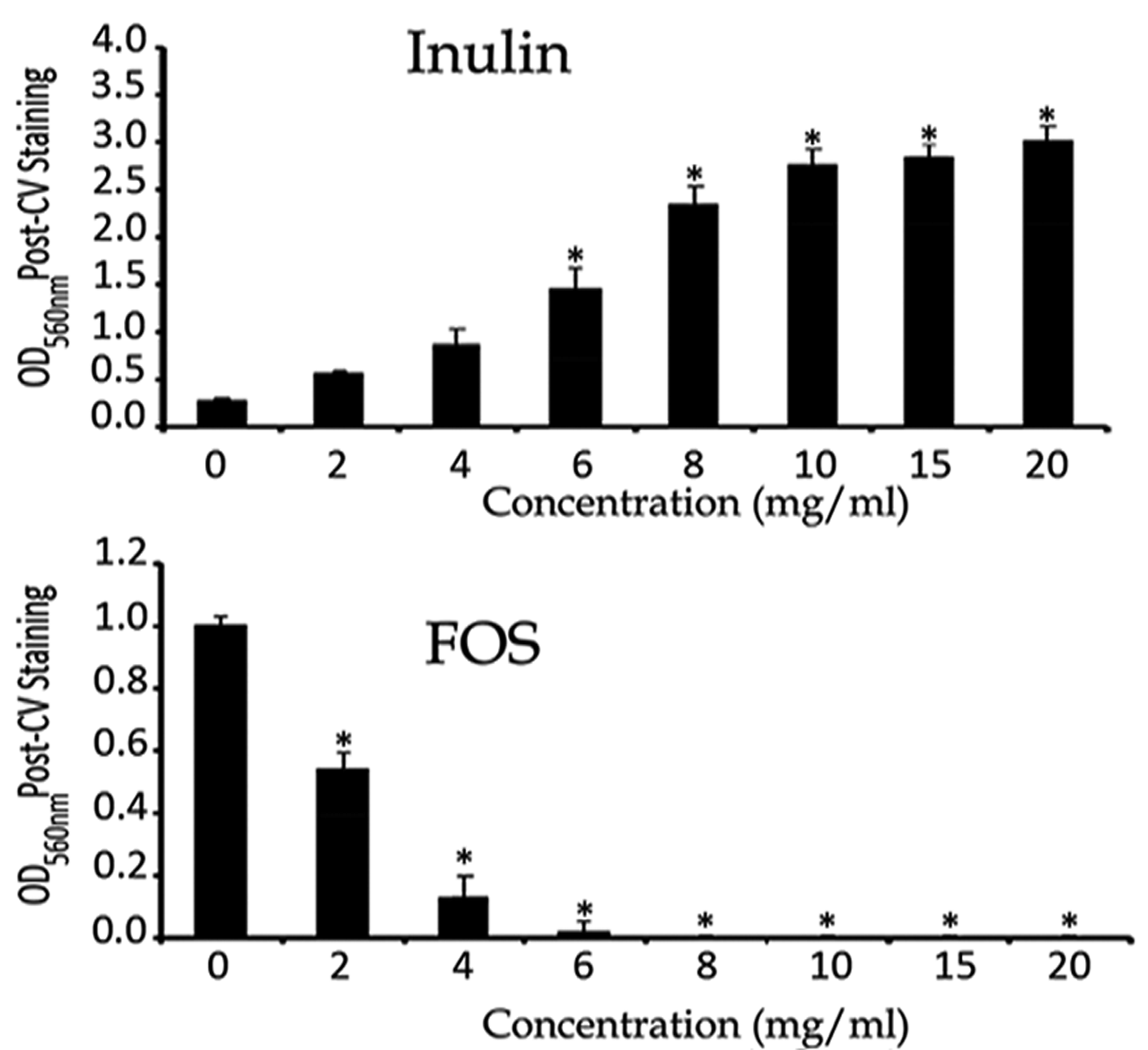

Supplement: Figure S1 — The OD at 560 nm of crystal violet (CV) stained and resuspended bacteria from biofilm are given. Shown are means and standard deviations with n = 3–6; *P<0.05 vs WT in the absence of inulin or FOS (ANOVA followed by least significance tests). The densitometric analysis of experiments is shown in Fig. 2A. (TIF) [file pone.0085772.s001.tif]

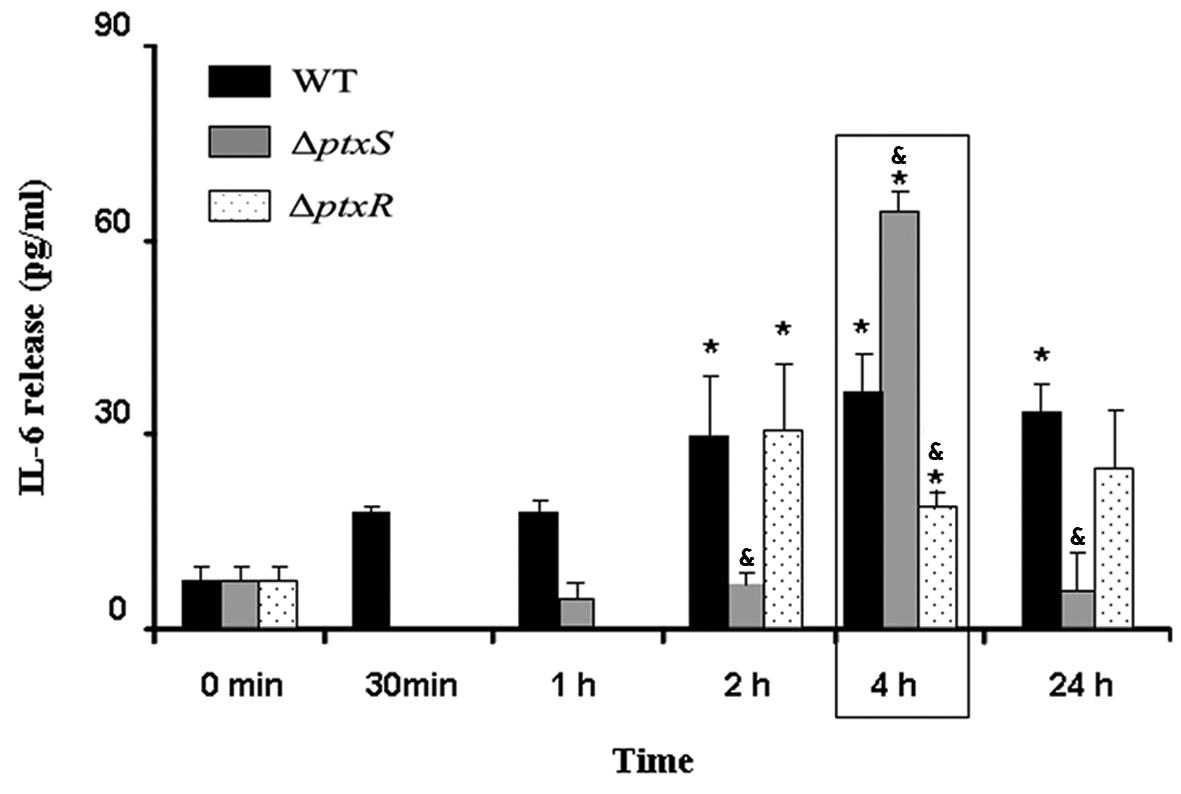

Supplement: Figure S2 — Measurement of interleukin-6 secretion of macrophages in the presence of WT P. aeruginosa PAO1 and its ptxS and ptxR mutants. The macrophage/bacteria ratio was of 1∶5. Experimental conditions involving incubation for 4 hours were subsequently used to assess the effect of FOS and inulin on interleukin secretion as reported in Fig. 4 and 7. Values are means ± s.e.m., n = 3; *P<0.05 vs macrophage without bacteria and &P<0.05 vs macrophage with WT (ANOVA followed by least significance tests). (TIF) [file pone.0085772.s002.tif]
